# Associations Between the Severity of Obsessive-Compulsive Disorder and Vocal Features in Children and Adolescents: Protocol for a Statistical and Machine Learning Analysis

**DOI:** 10.2196/39613

**Published:** 2022-10-28

**Authors:** Line Katrine Harder Clemmensen, Nicole Nadine Lønfeldt, Sneha Das, Nicklas Leander Lund, Valdemar Funch Uhre, Anna-Rosa Cecilie Mora-Jensen, Linea Pretzmann, Camilla Funch Uhre, Melanie Ritter, Nicoline Løcke Jepsen Korsbjerg, Julie Hagstrøm, Christine Lykke Thoustrup, Iben Thiemer Clemmesen, Kersten Jessica Plessen, Anne Katrine Pagsberg

**Affiliations:** 1 Department of Applied Mathematics and Computer Science Technical University of Denmark Copenhagen Denmark; 2 Child and Adolescent Mental Health Center Copenhagen University Hospital Mental Health Services Copenhagen Copenhagen Denmark; 3 Department of Applied Mathematics and Computer Science Technical University of Denmark Lyngby Denmark; 4 Centre for Functional and Diagnostic Imaging and Research Danish Research Centre for Magnetic Resonance Copenhagen University Hospital Amager and Hvidovre Denmark; 5 Department of Clinical Medicine Faculty of Health and Medical Sciences University of Copenhagen Copenhagen Denmark; 6 Center for Clinical Neuropsychology Children and Adolescents Rigshospitalet Copenhagen Denmark; 7 Division of Child and Adolescent Psychiatry Department of Psychiatry, Lausanne University Hospital, Centre Hospitalier Universitaire Vaudois University of Lausanne Lausanne Switzerland

**Keywords:** machine learning, obsessive-compulsive disorder, vocal features, speech signals, children, teens, adolescents, OCD, AI, artificial intelligence, tool, mental health, care, speech, data, clinical trial, validity, results

## Abstract

**Background:**

Artificial intelligence tools have the potential to objectively identify youth in need of mental health care. Speech signals have shown promise as a source for predicting various psychiatric conditions and transdiagnostic symptoms.

**Objective:**

We designed a study testing the association between obsessive-compulsive disorder (OCD) diagnosis and symptom severity on vocal features in children and adolescents. Here, we present an analysis plan and statistical report for the study to document our a priori hypotheses and increase the robustness of the findings of our planned study.

**Methods:**

Audio recordings of clinical interviews of 47 children and adolescents with OCD and 17 children and adolescents without a psychiatric diagnosis will be analyzed. Youths were between 8 and 17 years old. We will test the effect of OCD diagnosis on computationally derived scores of vocal activation using ANOVA. To test the effect of OCD severity classifications on the same computationally derived vocal scores, we will perform a logistic regression. Finally, we will attempt to create an improved indicator of OCD severity by refining the model with more relevant labels. Models will be adjusted for age and gender. Model validation strategies are outlined.

**Results:**

Simulated results are presented. The actual results using real data will be presented in future publications.

**Conclusions:**

A major strength of this study is that we will include age and gender in our models to increase classification accuracy. A major challenge is the suboptimal quality of the audio recordings, which are representative of in-the-wild data and a large body of recordings collected during other clinical trials. This preregistered analysis plan and statistical report will increase the validity of the interpretations of the upcoming results.

**International Registered Report Identifier (IRRID):**

DERR1-10.2196/39613

## Introduction

Obsessive-compulsive disorder (OCD) is a chronic, debilitating disorder, which can lower self-esteem, shorten life-expectancy, strain the family, and make it difficult to maintain friendships and attend school [1,2]. First-line treatment for moderate to severe OCD in youth (defined as individuals under age 18 years of age) is cognitive behavioral therapy (CBT) with exposure and response prevention (ERP) [3,4]. During exposure practice, the child tracks the level of distress caused by symptom-provoking stimuli across and within sessions. Here, distress refers to fear, disgust, discomfort, shame, embarrassment, and feelings of incompleteness or emptiness. Monitoring distress provides useful information to clinicians, who use it to plan exposures and help the patient remain mentally present with the exposure [5]. Distress levels also comprise one dimension of the gold-standard measure of symptom severity in OCD. When collected over time, distress can provide information about disease progression and improvement [6]. Frequent measures of distress are essential for understanding mechanisms of change in exposure-based therapies [5]. Self-rated distress may not be frequent enough to discover the processes responsible for therapeutic change, which has led some researchers to code videos of exposure sessions [7]. Behavioral coding is a time-consuming, costly process that is prone to inconsistency and not entirely immune to bias. Ideally, distress would be assessed objectively and affordably in a noninvasive manner.

Objective and automatic psychiatric assessments can be achieved by feeding vocal features into machine learning models. Speech patterns, tempo, volume, and intonation comprise an important part of the overall clinical impression that has been used to diagnose psychiatric disorders for at least 100 years [8]. Speech reflects changes in cognition, affect, motor characteristics, and physiology seen in psychiatric disorders [9]. Voice quality features capture information relating to voice creakiness, harshness, and breathiness [9]. Decreased formant frequencies observed in depressed and anxious speech may reflect dry mouth, decreased articulation, or motor coordination [9].

Vocal features have demonstrated promising results in machine learning for predicting the severity of psychiatric disorders and clinical improvement. A recent review summarized 127 studies that have used automatically extracted speech features to detect the presence or severity of psychiatric disorders [10]. The vocal fold features, jitter, and shimmer were found to be significantly elevated in adults with depression, anxiety, and OCD. Only one study has investigated vocal fold features in OCD. Results from 35 adults suggest that individuals with OCD have voices with more jitter, breathiness, hoarseness and speak at a lower rate than individuals without a psychiatric diagnosis [11]. Increased percent jitter and hoarseness have also been found in children (6-15 years old) with attention-deficit/hyperactivity disorder compared with healthy controls [12].

One major conclusion of the systematic review is that future studies should focus on linking vocal features to specific transdiagnostic problems, such as distress [10]. One study found that self-reported distress and electrodermal activity corresponded with vocal indicators of distress [13]. High levels of cortisol have also been linked to vocal indicators of distress [14]. Another study used audio recordings of 3- to 8-year-old children giving a speech under stressful conditions. The machine learning model classified children with and those without internalizing disorders using vocal features as input with 80% accuracy [15].

The aim of this paper is to increase the robustness of a planned study by documenting our analysis plan of a study with the following objectives: (1) to investigate the correspondence between activation-specific features and OCD diagnosis and severity [16] and (2) to adapt models trained on adult English speech to better align with the target population (Danish speech from children), thus obtaining improved indicators of OCD. In this paper, we also weigh our methodological decisions.

## Methods

### Participants and Setting

We will include audio recordings from a total of 64 youths aged 8 to 17 years (47 with OCD and 17 healthy controls). The audio recordings of diagnostic interviews stem from a large randomized clinical trial and case-control study of OCD, called TECTO [17]—TECTO runs at a public hospital in Denmark.

### Ethics Approval

**Figure 1:**
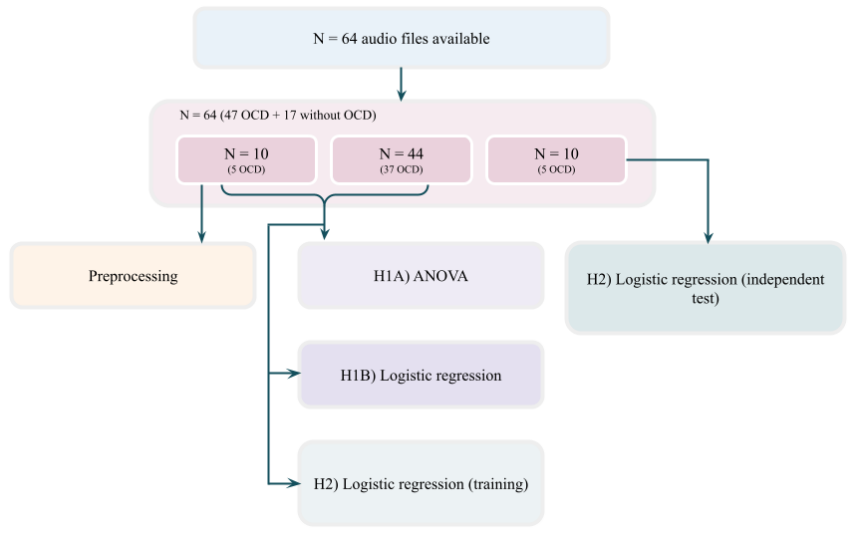
Flow diagram of audio data selection. OCD: obsessive-compulsive disorder.

TECTO and the planned analyses have been approved by the ethics committee of the Capital Region of Denmark (H-18010607). The selection of participants in the current study is depicted in Figure 1.

### Measures

Trained mental health professionals conducted clinical interviews before inclusion in TECTO to establish diagnoses and OCD severity in patients and rule out psychiatric diagnoses in controls.

#### Diagnostic Status

Trained mental health professionals used a semistructured clinical interview—the Kiddie Schedule for Affective Disorders and Schizophrenia (K-SADS)—to screen for and establish diagnoses in participants [18]. The K-SADS is designed to establish psychiatric diagnoses in youth between the ages of 6 and 18 years.

#### OCD Severity

Trained mental health professionals assessed the clinical severity of OCD using the Children’s Yale-Brown Obsessive Compulsive Scale (CY-BOCS) [19]. The CY-BOCS interview begins with a checklist of obsessions and compulsions to establish which symptoms have been present over the past week. On a scale from 0 to 4, clinicians rate 5 items on the severity of obsessions and 5 items on the severity of compulsions. Severity is rated on 5 dimensions: level of distress caused by the symptoms, functional interference, time consumed by symptoms, and resistance to and degree of control over symptoms. A total severity score is calculated by summing all 10 items on a scale from 0 to 40. Although CY-BOCS scores are continuous, a previous study of 815 youth between the ages of 4 and 18 years found the following cutoff scores to be consistent with global clinical severity ratings: 0-7, subclinical; 8-15, mild; 16-24, moderate; and 25-40, moderate-severe to severe [20]. A CY-BOCS score of ≥16 was required to be included in the study.

### Audio Data Source

**Figure 2:**
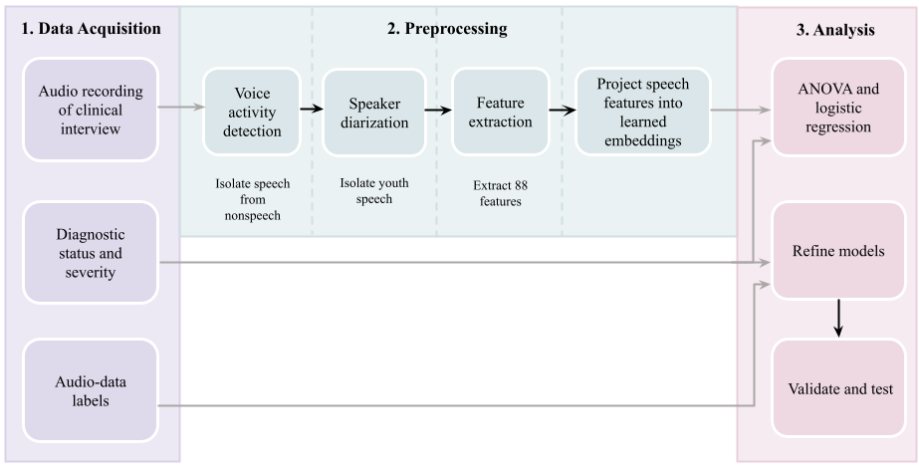
Overview of the data analysis process.

Samples of child speech will be taken from the K-SADS interviews. Owing to limited labeled data, we will use the first 10 minutes of the 30- to 90-minute interviews. Audio recordings were obtained using an on-camera microphone of a Sony video camera placed in various positions and in different rooms across observations. Data analysis is outlined in Figure 2.

### Statistical Analysis Plan

The planned study has two main objectives:

1. Test the usefulness of a previously learned latent model, composed of 2 dimensions representing a compressed vocal feature space guided by activation labels [16], as a marker for OCD diagnosis and severity.
2. Learn a new latent model, which we will propose as an improved candidate indicator of OCD severity.

Our first objective will be achieved through 2 statistical analyses, with the following hypotheses:

1. Objective 1 (H1): an ANOVA will be conducted to test the first research hypothesis (objective H1A) that there is an effect of diagnosis (OCD vs no psychiatric diagnosis) on the vocal feature latent model. With logistic regression (H1B), we will test hypothesis H1, which states that there is an effect of the vocal feature latent model on OCD severity classifications in moderate to extreme OCD cases, with the vocal feature latent model corrected for age and gender. This analysis will only include patients with OCD, and we will examine the classification accuracy of this model.
2. Objective 2 (H2): the second objective will be achieved through data labeling and machine learning modeling. Here, outcomes will include diagnostic status (OCD vs no psychiatric diagnosis) and OCD severity (CY-BOCS severity scores). We will validate the modeling using a leave-two-individuals-out cross-validation and prediction accuracies. Subsequently, we will assess the obtained results using an independent test. We will extract data from 10 of the youths’ audio samples post machine learning modeling for this purpose.

### Processing Pipeline

#### Preprocessing Audio and Annotations

**Figure 3:**
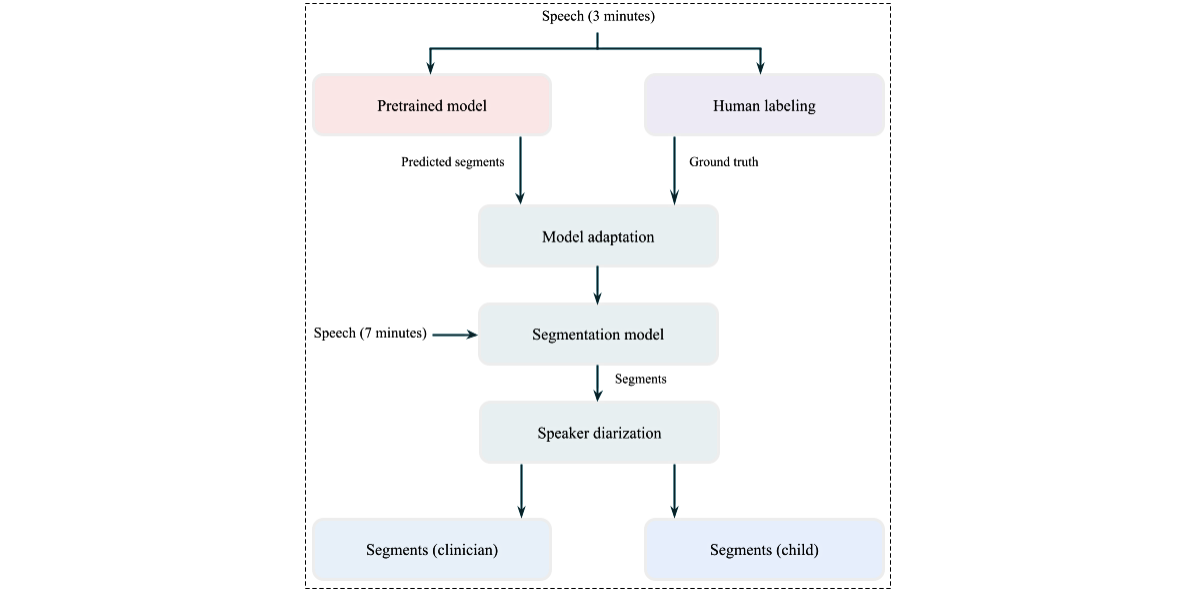
Flow diagram of the preprocessing pipeline.

The audio preprocessing system is illustrated in Figure 3. To effectively use the audio signals, the audio recordings must be segmented into shorter segments. Next, speech and nonspeech regions must be differentiated. To obtain speaker segments, the conversations will first be segmented into speech and nonspeech regions.

We will use pretrained voice activation and diarization models that will be fine-tuned with a few manually annotated ground-truth labels of speech and nonspeech samples from our data set [21]. We follow this approach to ensure a balance between resources spent in training a model while maintaining high accuracy in the obtained speaker segments. Following speaker segmentation of the audio recordings, only the audio segments corresponding to the youth are retained.

#### Pretrained Segmentation and Diarization Model

We used the vad-crdnn-libriparty pretrained model from SpeechBrain for speech detection [22] owing to its flexibility and modular structure, which is a favorable quality when applying models to clinical data owing to the tractability of errors. The pretrained model is based on a combination of convolutional and recurrent neural networks and a fully connected network. The model accepts audio segments as input and yields a posterior probability frame-level or segment-level posterior probability as output. Finally, a threshold is applied on the output posterior probability to classify the segments as speech and nonspeech.

We define a speaker segment to be an utterance of 2-4 seconds from a youth. The interviews will have a varying number of utterances. For a balanced analysis, we will randomly select 10 speaker segments per youth. Since the random selection of speaker segments may result in a high variance within the observations, we will also explore the feasibility of analyzing specific audio segments consistently over all speakers. These audio segments will be selected on the basis of insightful segments in the interview; for instance, questions in the interview associated with depression. In total, 10 interviews—5 with youth with OCD and 5 with those with no psychiatric diagnosis—have already been used to develop an appropriate method for speaker segmentation [21]. We will add the remaining 54 observations once we commence the described analysis.

#### Audio Features

From each speaker segment, we will extract the extended Geneva minimalistic acoustic parameter set [23], consisting of functionals of lower-level features. We will use the OpenSmile toolkit to extract these features and the resulting feature vector has a length of 88 [24].

The derived feature vector will be used as input to a neural network autoencoder model to obtain a latent model. The latent model is pretrained on English speech using the Interactive Emotional Dyadic Motion Capture data set and a semisupervised loss as previously described [16]. The latent model is a 2D latent space (*v*1 and *v*2) in a semisupervised denoising autoencoder with a reconstruction loss plus a loss based on the linear association between the activation labels and the latent space (the aim is a high association). Thus, to obtain *v*1 and *v*2, the 88 vocal features are projected into the latent space through the pretrained network. Previous analyses indicate that this latent space represents 2 dimensions of speech activation or intensity [16]. Sad and bored speech is characterized by low activation whereas elated and angry speech is characterized by high activation [25]. Thus, we assume that this latent space represents emotional intensity. We do not have an objective metric to evaluate this quantitatively using the current data and labels. Therefore, we will test this assumption in future work when more data labels are available. 

For the machine learning analyses, we will use the raw speech signals to learn new features.

### Statistical Models

Let *v*1 and *v*2 describe the vocal feature latent model adjusted for age and gender effects.

#### Statistical Models for Objective H1A

We will perform an ANOVA using a mixed effects regression model with the vocal features *v*1 and *v*2 as the outcome. We code the diagnosis, *Diagnosis*={0, 1} for no-OCD or OCD, respectively. The model for the *j*th vocal feature is as follows:

*v_ij_* = *μ_j_* + *α_j_*(*age_i_*) + *γ_j_*(*gender_ii_*) + *v_j_*(*age* × *gender*)*_i_* + *δ_j_*(*Diagnosis_i_*) + *y_j_*(*youth_i_*) + *ε_ij_* (1)

where *μ_j_* is a constant offset for the *j*th endpoint, *i* = 1, ..., 200, *y_j_* (*youth*_i_) ∼ *N* (0, *σ*^2^*_yj_* ), *ε_i_* ∼ *N* (0, *σ^2^_j_*), and the effects are mutually independent. Age, gender, and an interaction between age and gender are included as fixed effects to remove confounding effects from these variables. Diagnosis is included as a fixed effect and is our primary interest. The individual youth, denoted *youth*, is included as a random effect. *Diagnosis* is included as a fixed effect. In total, 10 repeated measures are available for each of the 64 youths included in this model.

With this analysis, we shall test the hypothesis that there is no effect of diagnosis; that is, *H*_0_ : *δ_j_* = 0, or equivalently that the means of the vocal features are equal for the OCD and no-OCD groups (when effects of age and gender are removed), at a 1.25% level of significance.

#### Statistical Models for Objective H1B

We will use a logistic regression with OCD severity categories moderate and severe-extreme as the outcomes. We denote the severity *s* = 0, 1. We will fit the following logistic regression model to the scores:


logit(si) = θ + b1
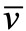
i + b2
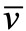
i + εi (2)


where *ε_i_* ∼ *N* (0, *σ*_2_*_j_*) and v*_j_* is the *j*th latent vocal feature adjusted for age and gender and averaged over the 10 repetitions for each youth, as described in the following.

This analysis aims to test the effects from vocal features on the severity scores through the null hypotheses that *b*_1_ = 0 and *b*_2_ = 0, which we will test at a 1.25% level of significance.

#### Adjusting for Confounding and Calculating Average Features

We expect age and gender to have an influence on the vocal features and would like to remove these confounding effects from the signals. We will exclude effects from age, gender, and age and gender interactions from the vocal features through a linear regression, as follows.

First, we will find the confounding effects using a linear regression model, which estimates age and gender contributions to vocal features as follows:



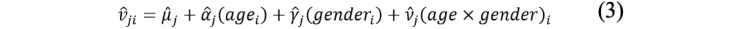



where, for the *i*th observations and *j*th vocal feature, *j* = 1, 2. The vocal features adjusted for age and gender are then as follows: 



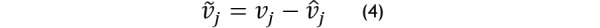



Finally, we will use the average vocal features over the 10 repetitions for the *k*th youth to obtain 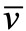 *_jk_* = Σ*iεyouth_k_ v_ji_* / 10.

#### Adjusting for Multiple Testing

We are interested in the null hypotheses associated with the effect of a diagnosis on the two dimensions in the latent model as well as the effects from the two adjusted latent dimensions on the severity scores. We will adjust our 4 *P* values using the Bonferroni method, and thus test each null hypothesis at a 5%/4=1.25% level of significance resulting in a family-wise error rate for the four tests of 5%.

#### Machine Learning Models for Objective 2

To improve the vocal features for possible use as biomarkers of OCD severity, we shall use the following methodology. First, 2 authors (SD and NLL) will annotate samples for activation and valence on a 5-point Likert scale with 1 indicating low activation and 5 indicating high activation. We shall only provide the annotators with one speech segment at a time in a blinded manner and in random order.

For the planned analyses, we will not use the valence labels, but the activation labels as these labels have shown promise in previous investigations [16]. We will use these labels as in our previous work [16]. The major advantages of labeling speech signals with activation scores include making labels for Danish speech and for children’s speech, and labeling speech of patients and controls. Thus, the labels provide added information as no open source data exist, thus supporting transfer learning wherever feasible.

Subsequently, we shall train the following machine learning models using the following annotations: variational and denoising autoencoders with semisupervised losses based on activation labels and a reconstruction error, plus a logistic regression or logistic regression loss in a neural network to obtain a classification model.

### Model Validation Strategies

We will use a leave-two-youth-out cross-validation, leaving 1 control plus 1 patient out for validation in each fold to tune the hyperparameters in our machine learning models and to choose between a simple logistic regression or a neural network with a logistic regression loss. To validate the results, we will use an independent test set of 10 interviews (5 with the patients and 5 with the controls) to evaluate the performance of these methods. These interviews will be transferred at the time of the independent test.

### Software

Data will be processed and analyzed using the most current and reliable version of Praat [26], Audacity [27], Python [28], R (R Core Team) [29,30], and OpenSmile [24].

## Results

### Statistical Report Model 1A

**Figure 4:**
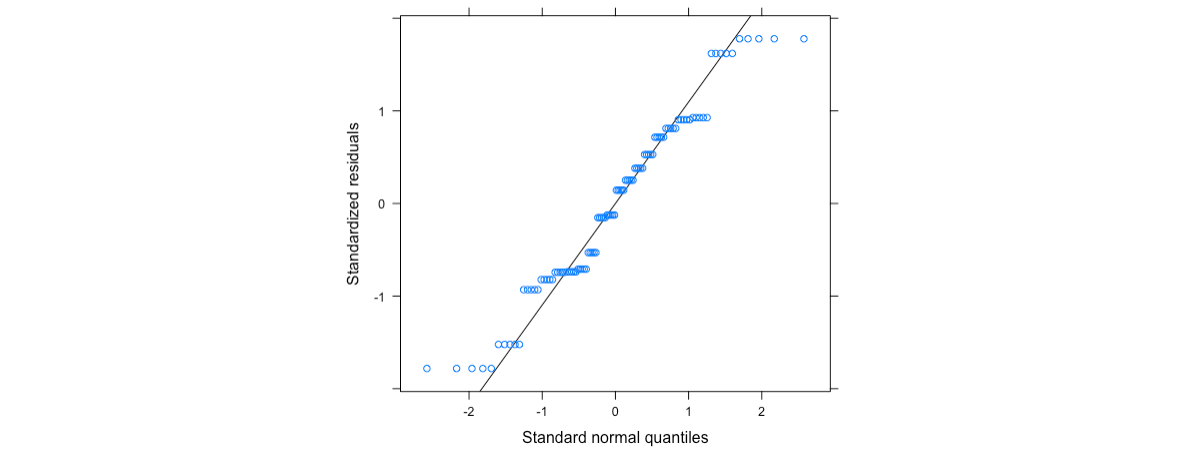
Quantile-quantile plot of residuals from model 1A with end point *v_j_*. Data are simulated and placeholders for results.

The statistical analysis in this section was performed with simulated data, simulated with the same structure as described above; that is, repeated measures from 10 individuals and with age and gender effects on the vocal feature latent model. The model 1A residuals are illustrated in Figure 4.

No strong effects were found, indicating that the assumptions have been violated. Thus, in this case, we will analyze the results from the model. A summary of the fixed effects is provided in Table 1 and one for the random effects is given in Table 2.

Finally, the CIs for all the parameter estimates are summarized in Table 3. We shall repeat this analysis for *v*1 and *v*2. We shall compare the *P* value [*Pr*(> |*t*|)] from the diagnosis to the 1.25% level of significance to asses if we can reject our null hypothesis of no effect from diagnosis. In the simulation, 2.92×10^–06^ is less than .0125; thus, we would reject the null hypothesis.

**Table 1:** Summary of fixed effects for *v_j_*. Data are simulated and placeholder for results.

Effect	Estimate	SE	*t* test	*df*	*P* [r(>|*t*|)] value
Intercept (*μ*)	1.16	0.12	9.54	4.99	2.15×10^–^^4^
Age (*α*)	0.49	0.01	51.23	4.99	5.36×10^–^^8^
Gender (*γ*)	0.83	0.13	6.16	4.99	1.64×10^–^^3^
Diagnosis (*δ*)	0.94	0.04	22.95	4.99	2.92×10^–^^6^
Age:gender (*ν*)	0.02	0.01	1.19	4.99	2.87×10^–^^1^

**Table 2:** Summary of simulated random effects *v_j_* with 100 observations and 10 groups (youth).

Groups	Variance	SD
Youth (*σ_y_*)	2.29×10^–^^3^	4.79×10^–^^2^
Residual (*σ_e_*)	2.33×10^–^^3^	4.83×10^–^^2^

**Table 3:** Simulated CIs for effects on *v_j_*.

Effect	Effects (%), CI	
	2.5%	97.5%
*σ_y_*	1.87×10^–^^2^	5.73×10^–^^2^
*σ_e_*	4.2×10^–^^2^	5.63×10^–^^2^
Intercept	9.71×10^–^^1^	1.34
Age	4.76×10^–^^1^	5.05×10^–^^1^
Gender	6.23×10^–^^1^	1.04
Diagnosis	8.74×10^–^^1^	9.99×10^–^^1^
Age:gender	–4.16×10^–^^3^	3.34×10^–^^2^

### Statistical Report Model 1B

**Figure 5:**
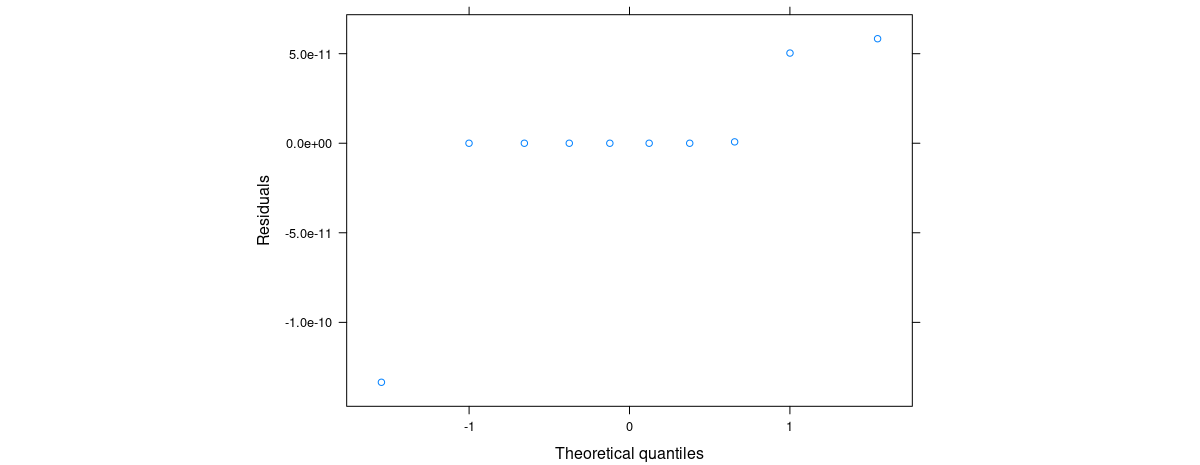
Quantile-quantile plot of surrogate residuals from model 1B with endpoint *v_j_*. Data are simulated and placeholders for results.

We shall first assess the assumptions of our model by examining the surrogate residuals in a quantile-quantile (q-q) plot (see Figure 5). In this case, the assumptions are violated as the residuals are not normally distributed. In case the assumptions are violated, we will not report on parameter estimates and their confidence intervals, as these will not be meaningful. However, we can still use the model for predictions.

**Figure 6:**
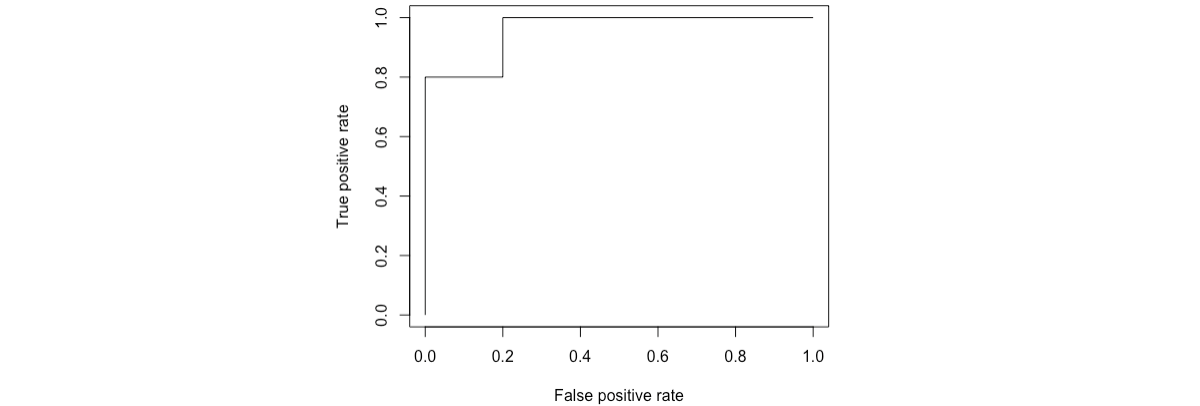
Receiver operating characteristic curve for the training data.

Figure 6 illustrates the receiver operating characteristic (ROC) curve for the training data, and the estimated classification accuracy is 80%. Additionally, we will report sensitivity, specificity, area under the curve, and a confusion matrix, similar to the next section.

If the surrogate residuals show an approximated normal distribution in the q-q plot, we will report parameter estimates, etc, as we have for model 1A.

### Statistical Report Model 2

**Figure 7:**
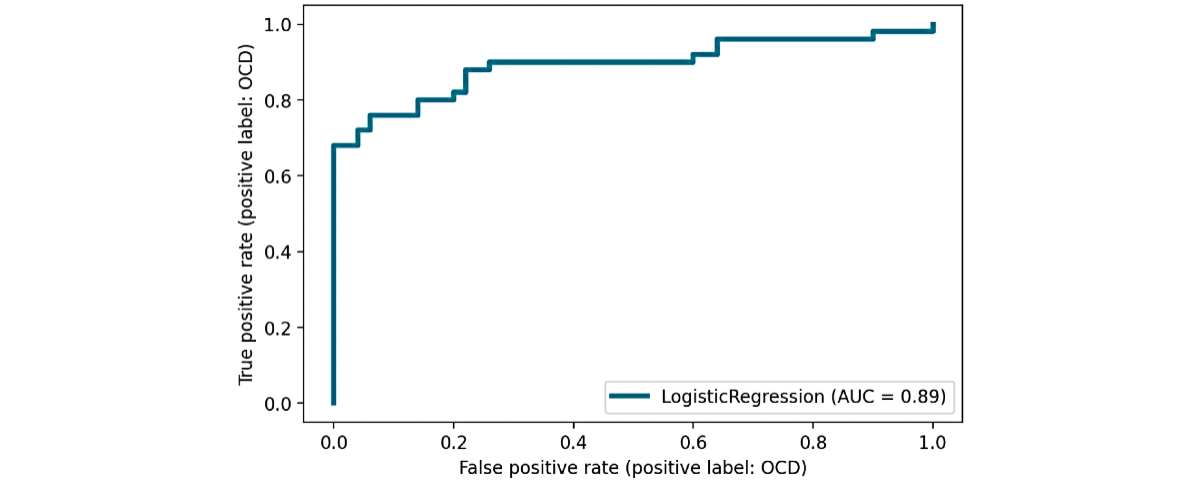
Receiver operating characteristic curve for the classification model. AUC: area under the curve; OCD: obsessive-compulsive disorder.

**Figure 8:**
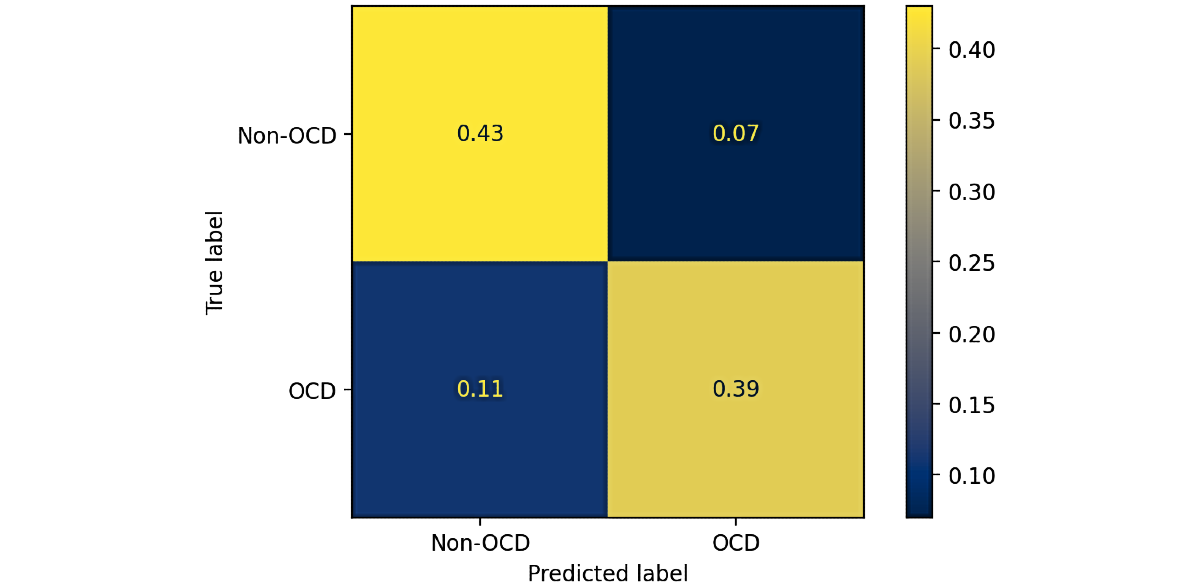
Confusion matrix for the classification model. OCD: obsessive-compulsive disorder.

The evaluation results of the machine learning model on simulated results are reported using the classification metrics in Table 4, the ROC curve in Figure 7 and the confusion matrix in Figure 8. The results will be presented for the validation data (the left-out youth) over the cross-validation to select hyperparameters and a model. We will also compare to the same measures for the training data to assess the amount of possible overfitting. Final results will be provided for the independent test data consisting of 10 youths.

**Table 4:** Simulated classification performance.

Evaluation metric	Value (normalized)
Accuracy	0.82
Sensitivity	0.78
Specificity	0.86
Area under the curve	0.89

## Discussion

### Expected Findings

In this paper, we describe the methods and analysis plan for testing the effects of OCD diagnosis and symptom severity on vocal features in the youth. We anticipate that we can improve the methods that model these putative associations. Through this work, we aim to obtain reliable transdiagnostic indicators of clinical severity from voice that would serve as valuable monitoring tools in psychiatry [31]. Additionally, the vocal indicators obtained from this work, in tandem with additional data modalities including video and semantics of the speech conversation, will be employed in multi-sensor modeling of OCD behavior in future work [32]. The results described in this analysis plan will be published in relevant scientific journals.

### Strengths

A major strength of the planned study is this documentation of the analysis plan prior to performing analyses. Documented a priori hypotheses prevent unscientific practices such as selectively reporting significant results [33]. Furthermore, diagnostic status and OCD severity levels were established by trained mental health professionals using gold-standard clinical interviews. Another strength of the planned study is that we will include gender in the models. Compared with male speech, female speech is marked by a higher pitch. A study found that gender-specific models were more accurate in detecting sadness and positive and negative affect than gender-independent models [34]. Depressed speech is marked by reduced pitch, whereas anxious speech is marked by increased pitch [10].

Another study found that pitch appears more important for detecting stress in men while Root Mean Square Energy appears more influential in detecting stress in women [35]. Thus, if training data sets are overrepresented by one gender, an automatic depression or anxiety severity rating algorithm may misclassify speech by other genders. We also attempted to strengthen our analyses by adding age to the models. The available latent model that we will use in our study of youth of a wide age range were trained on adult speech. With age, pitch and formant frequencies tend to decrease [36]. From childhood to adolescence, speech rate increases, and conveying emotions with prosodic cues, including pitch and timing, is a development feat [37].

### Limitations

The planned analyses have some foreseeable challenges. First, we plan to recruit a small sample. Preprocessing and data labeling, required for the planned analyses, are time-consuming tasks. Our small sample size increases the risk of type II errors. Machine learning techniques are more robust as we can improve models by adding more labeled data and testing on a new independent data set. Second, the audio samples that will be analyzed were collected for clinical purposes and not under optimal conditions for audio feature extraction and analysis (ie, using high quality microphones in a quiet environment). Thus, methods developed on these data will likely translate well to other naturalistic settings [38-41]. Finally, this study focuses on the associations among OCD diagnosis, severity, and vocal features in the child. In future work, we will study the effect of OCD diagnosis on vocal features while accounting for secondary diagnoses and investigate the influence of clinician’s vocal characteristics on the vocal features of the child.

### Conclusions

This predefined plan will limit bias in the interpretations and conclusions of the reported results of the future publication. If the results in the planned study are promising, this will be a step toward using vocal sensing to automate objective assessments and monitoring of severity of psychiatric disorders such as OCD.

## Abbreviations

CBT: cognitive behavioral therapy
CY-BOCS: Children’s Yale-Brown Obsessive Compulsive Scale
ERP: exposure and response prevention
K-SADS: Kiddie Schedule for Affective Disorders and Schizophrenia
OCD: obsessive-compulsive disorder
q-q: quantile-quantile
ROC: receiver operating characteristic

## Data Availability

We are not permitted to share voice samples or other personal identifying information. We will investigate the possibility of making an anonymized data set with extracted voice features available. If possible, we shall indicate where to obtain the data set with the published results.
